# Levels of gastrin-releasing peptide and substance P in synovial fluid and serum correlate with levels of cytokines in rheumatoid arthritis

**DOI:** 10.1186/ar1503

**Published:** 2005-02-07

**Authors:** Ola Grimsholm, Solbritt Rantapää-Dahlqvist, Sture Forsgren

**Affiliations:** 1 Department of Integrative Medical Biology, Section for Anatomy, Umeå University, Umeå, Sweden; 2 Department of Rheumatology, Umeå University Hospital, Umeå, Sweden

**Keywords:** cytokines, gastrin-releasing peptide, rheumatoid arthritis, substance P, TNF-α

## Abstract

It is well known that cytokines are highly involved in the disease process of rheumatoid arthritis (RA). Recently, targeting of neuropeptides has been suggested to have potential therapeutic effects in RA. The aim of this study was to investigate possible interrelations between five neuropeptides (bombesin/gastrin-releasing peptide (BN/GRP), substance P (SP), vasoactive intestinal peptide, calcitonin-gene-related peptide, and neuropeptide Y) and the three cytokines tumour necrosis factor (TNF)-α, IL-6, and monocyte chemoattractant protein-1 in synovial fluid of patients with RA. We also investigated possible interrelations between these neuropeptides and soluble TNF receptor 1 in serum from RA patients. Synovial fluid and sera were collected and assayed with ELISA or RIA. The most interesting findings were correlations between BN/GRP and SP and the cytokines. Thus, in synovial fluid, the concentrations of BN/GRP and SP grouped together with IL-6, and SP also grouped together with TNF-α and monocyte chemoattractant protein-1. BN/GRP and SP concentrations in synovial fluid also grouped together with the erythrocyte sedimentation rate. In the sera, BN/GRP concentrations and soluble TNF receptor 1 concentrations were correlated. These results are of interest because blocking of SP effects has long been discussed in relation to RA treatment and because BN/GRP is known to have trophic and growth-promoting effects and to play a role in inflammation and wound healing. Furthermore, the observations strengthen a suggestion that combination treatment with agents interfering with neuropeptides and cytokines would be efficacious in the treatment of RA. In conclusion, BN/GRP and SP are involved together with cytokines in the neuroimmunomodulation that occurs in the arthritic joint.

## Introduction

It has long been known that neuropeptides are secreted locally during immune responses [1], and that they are involved in vasodilation and plasma extravasation, that is, neurogenic inflammation [2,3]. It is furthermore known that inflammatory cells can produce neuropeptides [4,5]. The neuropeptide substance P (SP) has been extensively examined in normal joints and in rheumatoid arthritis (RA) during the past two decades. SP has known proinflammatory properties, enhancing the proliferation of rheumatoid synoviocytes [6] and inducing the release of the proinflammatory mediator prostaglandin E_2 _and destructive enzymes such as collagenase [6]. An increased level of SP has been observed in the synovial fluid from RA patients [7-11].

Another neuropeptide is gastrin-releasing peptide (GRP) [12], the mammalian homologue of bombesin (BN), a tetradecapeptide originally isolated from the skin of the frog *Bombina bombina *[13]. BN/GRP-like peptides affect several systems in mammals, such as satiety, thermal regulation, nociception, and the activation of the sympatho-adrenomedullary outflow [14,15]. We have recently shown that BN/GRP-like peptides are present in joint fluid in arthritis and that they are increased in amount in RA [11]. Several other neuropeptides, such as vasoactive intestinal peptide (VIP), calcitonin-gene-related peptide (CGRP), and neuropeptide Y (NPY), have also been found in synovial fluid from patients with RA [10,16]. VIP is a potent anti-inflammatory agent [17,18]. It therefore has a beneficial effect in collagen-induced arthritis (CIA) [17], inhibits the production of proinflammatory factors, including tumour necrosis factor (TNF)-α [19,20] and chemokines [21], and inhibits IL-2 production in stimulated murine T lymphocytes [22].

The proinflammatory cytokine TNF-α plays a central role in the pathogenesis of RA [23,24]. Increased concentrations of this cytokine in synovial fluid from RA patients have been reported in several studies (e.g. [25-27]). It has also been shown that TNF-α is expressed locally in joints of patients with RA and in inflamed joints in experimentally induced arthritis [28,29]. Increased concentrations of soluble TNF receptors (sTNFRs) p55 (sTNFR1) and p75 (sTNFR2) have been found in peripheral blood and synovial fluid from patients with RA [30,31]. These receptors can bind TNF-α and counteract its deleterious effects [30]. Serum concentrations of the two receptors have been correlated with RA disease activity [32]. The proinflammatory cytokine IL-6 is also found in elevated concentrations in the synovial fluid of RA patients [33]. The chemokine monocyte chemoattractant protein (MCP)-1 is a cytokine that attracts monocytes to a site of inflammation [34,35]. This chemokine has been suggested to be an important factor for the recruitment of leukocytes to the joints in RA and is also found in elevated concentrations in synovial fluid from RA patients [36-38].

Interactions between neuropeptides and cytokines are well known (e.g. [39-41]), and it has been suggested that regulatory neuroimmune pathways in the joints are important mechanisms modulating the expression of the arthritis [42]. However, there is limited information on the relations between neuropeptides and cytokines in the synovial fluid and serum from patients with RA. What is known is that SP and IL-6 concentrations correlate in synovial fluid from patients with RA [43]. Furthermore, the effect of SP on IL-1, IL-6, and TNF-α production in RA was recently examined [44]. CGRP has been found to reduce the production of IL-6 and MMP-2 in RA synovial cells *in vitro *[18]. To the best of our knowledge, there is no information at all on the occurrence of a possible relation between BN/GRP and cytokines in arthritic patients.

The lack of information on relations between neuropeptides and cytokines in RA hampers the understanding of whether neuropeptide-modifying agents, in addition to cytokine antagonists such as TNF-α blockers, might be useful in the treatment of RA. New information has led to suggestions that treatments interfering with neuropeptides should be tested in combination with pre-existing treatments [45]. A number of neuropeptide receptor agonists and antagonists produced during recent years have been found to have effects in other situations [46-49]. Actually, using an animal model of RA, collagen-induced arthritis, it was shown that VIP affected the Th1/Th2 balance by inhibiting the synthesis of Th1 cytokines and inducing the synthesis of Th2 cytokines [17]. Also, the results of recent studies *in vitro *on cells of human origin have suggested that modulation of the effects of neuropeptides may be a new strategy for the treatment of arthritis [50,51]. In the present study, we therefore aimed to investigate the relation between the concentrations of three cytokines (TNFα, IL-6, and MCP-1) and several neuropeptides (BN/GRP, SP, NPY, VIP, and CGRP) in the synovial fluid from patients with RA, stratified for early and long-standing disease. We also studied the concentrations of sTNFR1 in relation to these neuropeptides in serum. We found that BN/GRP and SP showed correlations with the proinflammatory cytokines in the group with long-standing RA.

## Materials and methods

### Patients and material

Thirty-five consecutive patients (25 women and 10 men) with RA according to the criteria of the American Rheumatism Association [52], having active inflammation of the knee joints, were included in the study. The patients were divided into two groups, depending on their disease duration. Patients were defined as having 'early RA' (*n *= 7; mean age, 51 years) or 'long-standing RA' (*n *= 23 to 28; mean age, 59 years). Median time when sampling of blood and synovial fluid for the early RA group was 8.0 months from onset of symptoms (interquartile range = 5.6 months); the median duration from onset of symptoms until diagnosis was 6.0 months. The early RA group were thus those patients who had had RA for less than approximately 12 months, and the patients in the long-standing RA group were those patients whose disease had lasted for more than a year.

The synovial fluid was aspirated according to the method of Dixon and Emery [53]. The aspirates were centrifuged, and the leukocytes were removed. The samples were frozen and stored at -80°C until assay. Blood samples were also collected concurrently from patients whose synovial fluid was examined: patients with early RA (*n *= 4) or with long-standing RA (*n *= 22). The sera were frozen and stored at -80°C until assay. The erythrocyte sedimentation rate (ESR) was measured for all the patients.

Synovial fluid (*n *= 2 to10) and serum (*n *= 2 to 11) was also obtained from healthy controls (mean age, 39 years). The samples were collected, frozen, and stored in the same way as the material from the RA patients.

The study protocol was approved by the Ethical Committee at the Faculty of Medicine, Umeå University, and the experiments were conducted according to the principles expressed in the Declaration of Helsinki.

### Enzyme-linked immunosorbent assay/enzyme immunoassay

The concentration of TNF-α in joint fluid was determined using a Human ELISA TNFα kit (Pierce-Endogen, Rockford, IL, USA) in accordance with the instructions from the manufacturer. The detection limit in this particular assay was cited as 2 pg/ml. The receptor level of sTNFR1 in serum was measured using a Human Quantikine sTNF R1 kit (R&D Systems, Wiesbaden-Nordenstadt, Germany). The minimum detectable level for this assay was 0.77 pg/ml. The concentrations of MCP-1 and IL-6 were determined with Human ELISA kits (Pierce-Endogen). The minimum detectable concentrations for these assays were <10 pg/ml and <1 pg/ml, respectively. The concentrations of the neuropeptides BN/GRP and SP (in serum) and of the neuropeptides CGRP, NPY, and VIP (in joint fluid and serum) were measured using Enzyme Immunoassay kits (Phoenix Pharmaceuticals, Belmont, CA, USA) in accordance with the supplier's assay instructions. The detection limits for the neuropeptides were as follows: BN/GRP, 300 pg/ml; CGRP, 230 pg/ml; NPY, 130 pg/ml; SP, 90 pg/ml; and VIP, 80 pg/ml.

### Radio-immunoassay

For determining concentrations of BN/GRP and SP in joint fluid, RIAs were performed. These conform to the analyses described and presented in a previous study [11]. The concentrations of BN/GRP-like material in joint fluid were determined using a ^125^I-RIA kit (Peninsula Laboratories Inc, San Carlos, CA, USA) in accordance with the manufacturer's instructions. The detection limit in this particular assay was cited as 10 pg/ml. For the SP assay, a ^125^I-RIA kit (Eurodiagnostica, Malmö, Sweden) was employed. The sensitivity of the assay was 2.5 pg/ml.

### Statistics

Differences between the continuous data for concentrations of the investigated substances in the two subject groups (early RA, long-standing RA) were analysed using the Mann–Whitney *U *test. Correlation analyses were performed with the Spearman rank correlation test. *P *values <0.05 were considered significant. Because the amounts of synovial fluid and serum from healthy controls varied, no statistical analysis was performed in this case.

Factor analysis, that is, explorative data analysis, was also performed to find patterns among the measured variables. In this case, because of the number of samples available, the pattern for long-standing RA was evaluated. Factor loadings >0.3 were considered. The factor analyses were performed by SPSS with Varimax, with Kaiser normalisation as a rotation method. For the statistical calculations, values below the detection limit were set to 50% of the detection limit and values beyond the highest standard point were set to the highest linearly detectable concentration.

## Results

### Cytokines in synovial fluid

**Figure 1:**
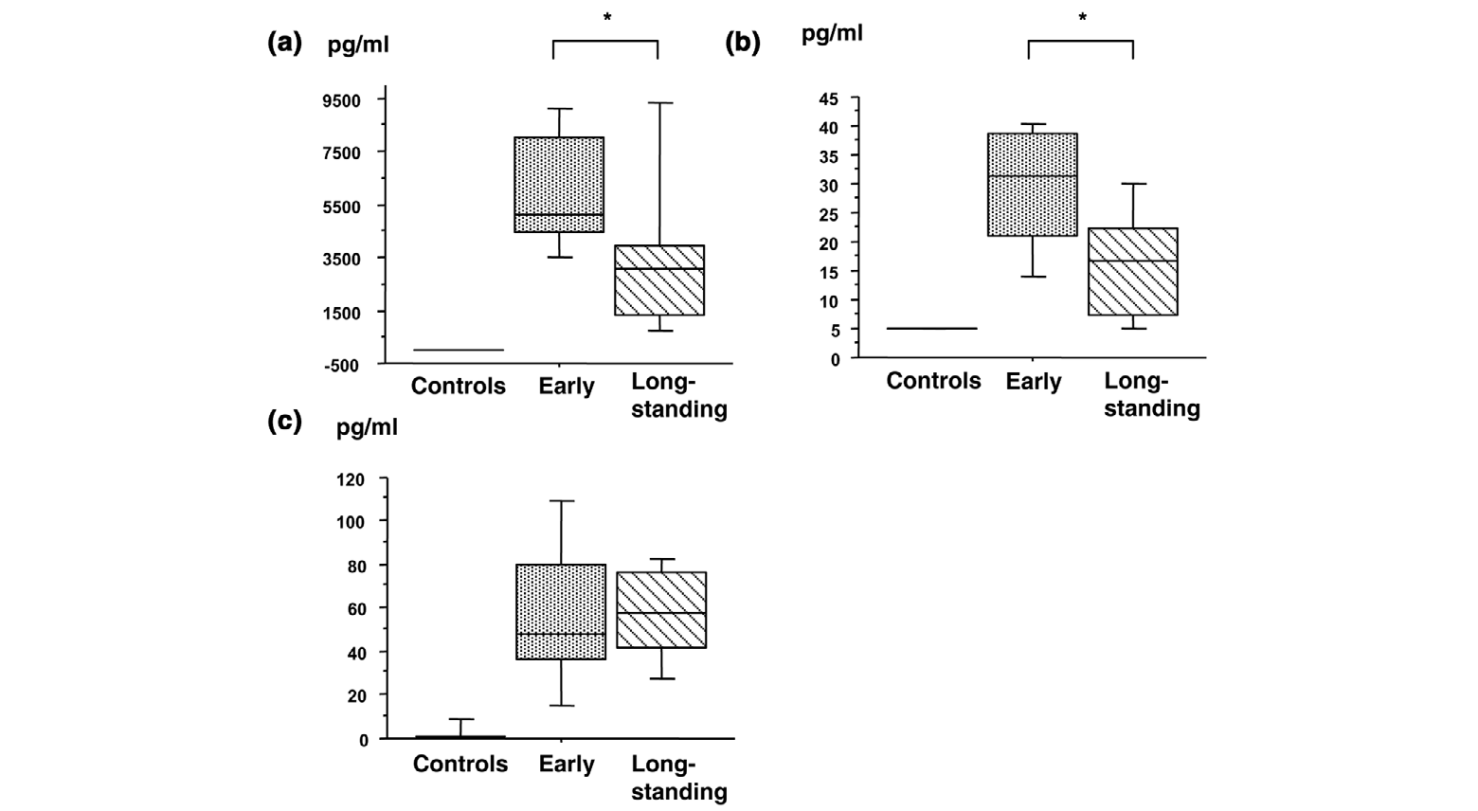
Box-whisker plots showing concentrations in synovial fluid for rheumatoid arthritis and healthy controls. **(a) **IL-6; **(b) **bombesin/gastrin-releasing peptide; **(c) **substance P. Boxes show the 25th and 75th percentiles; horizontal lines in boxes show the medians; whiskers show the 10th and 90th percentiles. *P < 0.05.

Concentrations of immunoreactive TNF-α in synovial fluid were detected in most of the patients (approximately 70%). Table 1 shows medians and interquartile ranges of the concentrations found. No significant differences were found between the two disease groups (Mann–Whitney test). The concentrations of immunoreactive IL-6 in synovial fluid were detected in 95% of the patients with RA. Medians and interquartile ranges are shown in Table 1. The patients with early RA had significantly higher concentrations of IL-6 than the other group (*P *< 0.05) (Fig. 1a). The chemoattractant cytokine MCP-1 was detected in all patients, (see Table 1). No significant differences were found between the two groups for MCP-1. Values found for control patients are shown in Table 1.

### Neuropeptides in synovial fluid

The neuropeptides BN/GRP, CGRP, NPY, SP, and VIP were detected in synovial fluid in 70 to 95% of the patients (Table 1). A statistically significant difference between the two groups, patients with early and those with long-standing RA, was found only for BN/GRP (*P *< 0.05) (Fig. 1b). SP and BN/GRP values in healthy controls were significantly lower than in the two disease groups (*P *< 0.001) (Fig. 1b,c). The values found for control patients are shown in Table 1.

### sTNFR1 in serum

**Figure 2:**
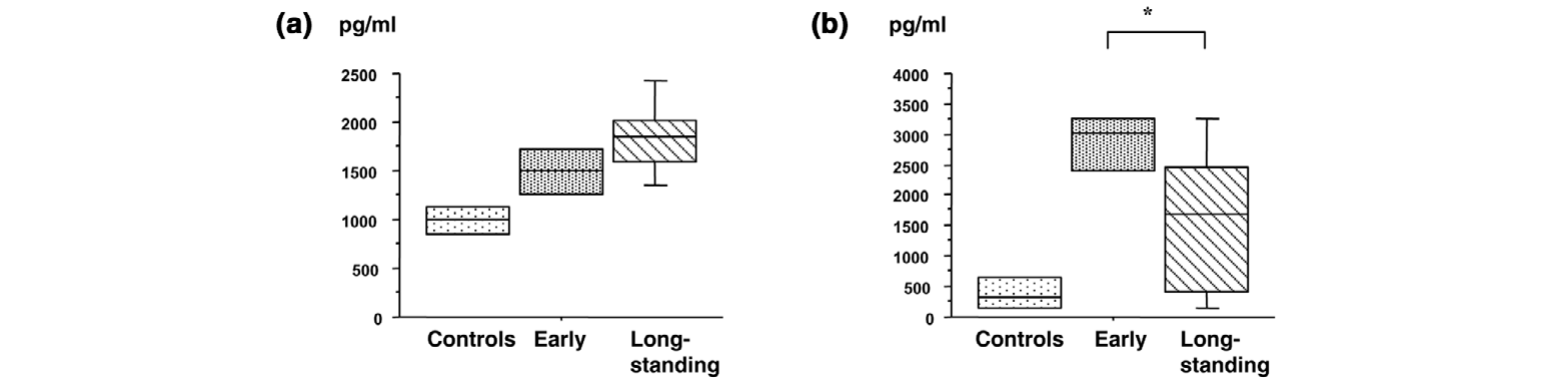
Box–whisker plots showing concentrations in serum for the patients with rheumatoid arthritis and healthy controls. **(a) **Soluble tumour necrosis factor receptor 1; **(b) **bombesin/gastrin-releasing peptide. Boxes show the 25th and 75th percentiles; horizontal lines in boxes show the medians; whiskers show the 10th and 90th percentiles. *P < 0.05.

Soluble TNFR1 was measurable in sera from all subjects (Table 2; Fig 2a.) Differences between the two disease groups were close to significance (*P *< 0.09).

### Neuropeptides in serum

The concentrations of the neuropeptides BN/GRP, CGRP, NPY, SP, and VIP in sera were measurable in 85 to 100% of the patients (Table 2). Statistically significant differences between the two groups of patients, those with early and those with long-standing RA, were found for BN/GRP (*P *< 0.05) (Fig. 2b) and for SP (*P *< 0.05). The values found for control patients are shown in Table 2.

### Correlations between cytokines and neuropeptides in synovial fluid

**Figure 3:**
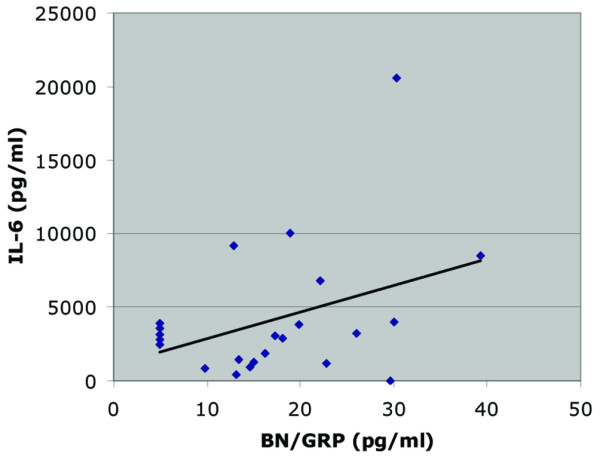
Scatter plot, with linear regression line, showing correlation between BN/GRP and IL-6 in synovial fluid. Spearman's rank correlation coefficient (*r*_s _= 0.38; *P *< 0.05) measured, using RIA and ELISA, in synovial fluid from patients with long-standing RA. BN/GRP, bombesin/gastrin-releasing peptide; RA, rheumatoid arthritis.

Spearman rank correlations between the concentrations of cytokines and those of the five neuropeptides were calculated. There was a correlation between IL-6 and BN/GRP-like peptides in long-standing RA but not in early RA (Fig. 3). There was also a correlation between TNF-α and BN/GRP-like peptides in long-standing RA (r_s _= 0.40; *P *< 0.05), but not in the early RA group.

Factor analysis performed on the long-standing RA group yielded four factors with eigenvalues >1, explaining 78% of the total variation between the measured variables (Table 3). SP, BN/GRP, and IL-6 grouped together, with the larger loadings on BN/GRP and on IL-6 (Table 3) in the group with long-standing RA. There was also an interrelation between SP, TNF-α, and MCP-1. None of the other three neuropeptides (NPY, CGRP, and VIP) showed any correlation with any of the cytokines. The ESR grouped together with three neuropeptides (BN/GRP, SP, and CGRP), with the largest loadings on SP and on the ESR.

### Correlations between sTNFR1 and neuropeptides in serum

**Figure 4:**
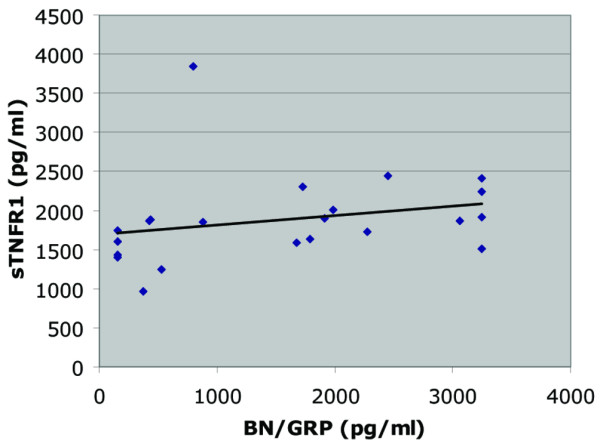
Scatter plot, with linear regression line, showing correlation between BN/GRP and sTNFR1 in serum. Spearman's rank correlation coefficient (r_s _= 0.61; *P *< 0.05), measured, using ELISA, in serum from patients with long-standing RA. BN/GRP, bombesin/gastrin-releasing peptide; RA, rheumatoid arthritis; sTNFR1, soluble TNF receptor 1.

Strong correlations on the Spearman rank test (r_s _= 0.61; *P *< 0.05) between concentrations of BN/GRP and those of sTNFR1 were found in the group with long-standing RA, but not in that with early RA (Fig. 4). No correlations were found between sTNFR1 concentrations and the concentrations of the other neuropeptides.

Factor analysis performed on the group with long-standing RA yielded three factors with eigenvalues >1, explaining 81% of the total variation between the measured variables (Table 4). It can be seen here that BN/GRP grouped together with sTNFR1 and CGRP. None of the other neuropeptides showed any correlation with sTNFR1. It was also seen that the ESR grouped together with sTNFR1.

### Correlations between the neuropeptides in synovial fluid

Spearman rank correlations showed that the concentrations of NPY in synovial fluid correlated with those of SP (*r*_s _= 0.41; *P *< 0.05), VIP (*r*_s _= 0.69; *P *< 0.01), and CGRP (*r*_s _= 0.52; *P *< 0.05). VIP also correlated with CGRP (*r*_s _= 0.7; *P *< 0.001).

Factor analysis showed that all five neuropeptides grouped together, with the larger loadings on VIP, CGRP, and NPY (Table 3).

### Correlations between the neuropeptides in serum

Spearman's rank correlations showed that the concentrations of BN/GRP in serum correlated with those of CGRP (*P *< 0.01; *r *= 0.70), VIP (*P *< 0.05; *r *= 0.51), and SP (*P *< 0.01; *r *= 0.58). Concentrations of SP also correlated with those of CGRP (*P *< 0.01; *r *= 0.54) and VIP (*P *< 0.001; *r *= 0.83). Furthermore, the concentrations of VIP correlated with the those of CGRP (*P *< 0.01; *r *= 0.54).

All the neuropeptides grouped together in the factor analysis on serum (Table 4).

## Discussion

In the present study, the relations between various neuropeptides and cytokines in joint fluid and serum of RA patients were examined. Although there were, to a certain extent, correlations between the various neuropeptides examined, a major finding was that BN/GRP and SP were the neuropeptides that correlated most clearly with the cytokines, with BN/GRP showing the highest degree of correlation. We stress that the occurrence of possible relations were sought extensively, using both Spearman correlation analyses and factor analysis. We also observed that the concentrations of BN/GRP and SP in synovial fluid grouped together with the ESR. These observations suggest that BN/GRP and SP are connected to inflammation in the joint. Comparison with the values obtained from healthy controls showed clearly that in the patients in the long-standing RA group, there were increases in concentrations of the cytokines examined, as well as in concentrations of SP and BN/GRP. It is well established that TNF-α is undetectable in synovial fluid from healthy controls [23,27].

CGRP, NPY, SP, and VIP have all been shown to affect cytokine production/release from various immune cells [39,40,44,54]. For example, SP can modulate the release of IL-1, IL-6, and TNF-α from human blood monocytes [39]. The stimulatory effect of SP on these proinflammatory cytokines was due to an augmented secretion from the immunocompetent cells [41]. SP and VIP have opposite roles concerning the regulation of TNF-α production, VIP inhibiting the production of TNF-α from, for example, microglia, and SP increasing the TNF-α production from mast cells and other types of inflammatory cells [40,41]. The impact of neuropeptides on cytokine production and release in RA is not well known. However, in a recent study it was shown that CGRP, NPY, SP and VIP could affect the IL-1β, TNF-α, and IL-6 production by peripheral whole blood cells from RA patients [44]. In the present study, we searched for possible correlations of TNFα, IL-6, and MCP-1 with all neuropeptides included in the analyses of the synovial fluid, but no correlations were found concerning VIP, NPY, or CGRP. The situation was the same for correlation matrices between sTNFR1 and these three neuropeptides in serum.

Because IL-6 was found in elevated concentrations in the synovial fluid of RA patients [33,55], the importance of this cytokine in RA and other diseases has been thoroughly investigated. It has been shown that IL-6 regulates acute-phase proteins such as C-reactive protein and haptoglobin [33,55]. Furthermore, a humanised anti-IL-6 receptor antibody has been found to inhibit IL-6 significantly and to improve the signs and symptoms of RA [56]. In the analysis of synovial fluid in the present study, we found that BN/GRP and SP grouped together with IL-6, with the strongest loadings on BN/GRP and on IL-6. This is one of the major findings leading to the interpretation that these two neuropeptides correlate more closely than the others with disease activity in RA. In the synovial fluid of patients with long-standing RA, there was also a correlation between BN/GRP and TNF-α. As it is well known that TNF-α and other cytokines may occur in insufficient concentrations in serum (e.g. [57]), we analysed sTNFR1 in serum and found that the receptor concentrations correlated with concentrations of BN/GRP on the Spearman's rank analysis and that they grouped together on the factor analysis.

It has long been discussed as to whether SP is clinically significant in RA, and accordingly whether SP-receptor antagonists block joint inflammation (e.g. [58,59]). In early studies, it was shown that intra-articular injections of SP increased the severity of the arthritis [60]. This substance was also early shown to facilitate the production and release of proinflammatory mediators such as prostaglandin E_2 _and destructive enzymes such as collagenase [6]. In more recent studies, intra-articular injection of SP was found to lead to endothelial-cell proliferation [61]. The overall role of SP in RA has recently been extensively discussed and it was concluded that the therapeutic potential of SP receptor (neurokinin-1 receptor) antagonists should not be ignored [45].

SP is present in the nerve fibres of the synovium [62]. However, both up- [63] and down- [64] regulation of SP expression has been noted for arthritic joints. In accord with these observations, up- and down-regulations of neuropeptide expressions occur in relation to the activity of inflammatory disease in other tissues [65,66]. A reason for decreased magnitude of SP in the nerve fibres of joint capsules may be the occurrence of a local release of SP, leading to a depletion of SP-containing nerve fibres [67-69]. There are also diverging observations concerning the occurrence of increases or decreases of SP concentrations in the synovial fluid of arthritic patients (cf. [7,10,11,16,70-72]). Taking into consideration that neuropeptide and cytokine concentrations may vary depending upon the stage of arthritis for the patients examined and that differences in actual neuropeptide concentrations in serum between different patients should be interpreted with caution, an advantage of the present study is that it focuses on intraindividual comparisons of neuropeptide and cytokine concentrations.

It is not clear whether BN/GRP-like peptides are present in joint innervation, or how the release of the peptides into the synovial fluid occurs. An interesting observation is that BN/GRP-like peptides have been detected in human chondrocytes [73]. In any case, BN/GRP-like peptides are well known to have trophic and growth-promoting effects in various parts of the body. Thus, these peptides have paracrine and autocrine trophic effects in certain cancers, such as small-cell lung cancer and colon cancer [74-76], and are likely to have trophic effects on the salivary glands after irradiation [77] and to promote proliferation of fetal chondrocytes [78]. Furthermore, GRP receptors have morphogenetic properties for colon cancer cells, these being mediated via focal adhesion kinase [79]. The expression of BN/GRP-like peptides in the spinal cord is influenced by glucocorticoids [80], which may be of interest for the understanding of the effects of treatments of RA patients, many of whom are treated with glucocorticoids. Concerning inflammation, it has been shown that BN/GRP-like peptides ameliorate burn-induced intestinal inflammation by preventing the sequestration of neutrophils in the injured tissue [81] and that these peptides presumably are mediators of inflammatory reactions during pulmonary inflammation [82]. Furthermore, BN ameliorates colonic damage in experimental colitis [83], which suggests that the peptide may be involved in restoring intestinal damage in inflammatory bowel disease. Treatment with BN/GRP has also been found to have antiulcerogenic and anti-inflammatory actions, related to effects on chronic gastric ulcers and burn-induced and colitis-induced gut injury [84]. Furthermore, topical use of GRP has been found to promote cutaneous wound healing [85].

The present study is relevant because neuropeptides have been suggested to be efficacious antiarthritic drugs [17,50,51]. Thus, VIP was shown to be effective as an anti-inflammatory agent *in vivo *for collagen-induced arthritic mice [17,86]. Furthermore, a recent study on human cells *in vitro *showed that VIP can down-regulate the expression and synthesis of chemokines in synovial tissue cells and fibroblast-like synoviocytes from RA patients and that VIP inhibits the production of TNF-α after TNF-α stimulation [51]. The results obtained by Foey and collaborators [50] show that the studied neuropeptide (VIP) is not on its own a useful therapeutic agent in the treatment of RA but may be useful in combination with phosphodiesterase inhibitor. In line with this idea, it has frequently been suggested that various types of combination treatments might be effective in diseases such as RA. It was initially suggested that a combination therapy targeting two cytokines instead of one would be the treatment of choice for RA [87] and other autoimmune diseases [88]. However, in a recent clinical trial it was observed that a combination therapy with a recombinant IL-1 receptor antagonist and polyethylene-glycol-conjugated sTNFRI (etanercept) did not result in an improvement of the clinical factors of RA in comparison with etanercept alone [89]. Alternative combination treatments might in the future include treatments targeting cytokine and neuropeptide. In line with this idea, it was recently suggested that SP-receptor (neurokinin-1 receptor) antagonists might be useful in combination with pre-existing treatments in RA [45]. In a recent study, a combination of common chemotherapy and immunotherapy targeting BN/GRP-receptor with a synthetic BN/GRP antagonist was found to enhance the killing of small-cell lung cancer cells [90].

As discussed above, there is indeed evidence showing that neuropeptides can modify the secretion of proinflammatory cytokines from immunocompetent cells. That includes the situation for peripheral blood cells from patients with RA [44]. As seen in the present study, BN/GRP and SP are the peptides that show correlations with the inflammatory cytokines in RA. Thus, we tentatively suggest that the increased concentrations of BN/GRP and SP may stimulate cytokine production from immunocompetent cells in RA. The findings that neurokinin-1 receptor is not only detectable on immunocompetent cells, but also on rheumatoid synoviocytes [91] and on endothelial cells in synovitis [61], strengthen the suggestion that SP plays an important role in the rheumatoid joint. In future studies, it would therefore be of interest to investigate whether BN/GRP and its receptor are active during the rheumatic process, and whether treatments interfering with the effects of BN/GRP or SP or both, possibly in parallel with medications affecting cytokine effects, are of value in RA.

## Conclusion

The neuropeptides that showed correlations with the proinflammatory cytokines in the patient material examined were BN/GRP and SP, particularly BN/GRP. The observations give new insight into further studies of neuropeptide/cytokine interrelations in RA.

## Abbreviations

BN/GRP = bombesin/gastrin-releasing peptide; CGRP = calcitonin-gene-related peptide; ELISA = enzyme-linked immunosorbent assay; ESR = erythrocyte sedimentation rate; GRP = gastrin-releasing peptide; IL = interleukin; MCP-1 = monocyte chemoattractant protein-1; NPY = neuropeptide Y; RA = rheumatoid arthritis; RIA = radio-immunoassay; SP = substance P; sTNFR1 = soluble TNF receptor 1; Th = T helper (cells); TNF = tumour necrosis factor; VIP = vasoactive intestinal peptide.

## Competing interests

The author(s) declare that they have no competing interests.

## Authors' contributions

OG participated in the design of the study, carried out the immunoassays, and performed the statistical analysis. SF participated in the design, coordinated the study, and took part in the statistical analysis. OG and SF drafted the manuscript. SRD was responsible for collection of the samples, participated in the statistical analysis, contributed to the coordination of the study, and took part in discussions concerning the manuscript. All authors read and approved the final manuscript.

## Figures and Tables

**Table 1:** Cytokine and neuropeptide concentrations in synovial fluid

Substance	Early RA (pg/ml)	Long-standing RA (pg/ml)	*P*	Controls (pg/ml)
*Cytokine*				
TNF-α	8.0^a^	7.0^c^	ns	nm
	(1.0–47)	(1.0–24)		
IL-6	5100^b^	3100^d^	<0.05	nd^f^
	(4500–8000)	(1300–3900)		
MCP-1	1200^b^	1500^d^	ns	620^f^
	(810–1800)	(1200–3000)		
*Neuropeptide*				
BN/GRP	31^a^	17^c^	<0.05	nd^g^
	(21–39)	(7.0–23)		
CGRP	580^b^	450^d^	ns	nd^f^
	(160–1500)	(230–960)		
NPY	190^b^	260^e^	ns	180^f^
	(65–320)	(170–500)		
SP	48^a^	58^c^	ns	nd^g^
	(36–80)	(42–77)		
VIP	150^b^	300^d^	ns	nm
	(40–200)	(55–630)		

Concentrations were measured by ELISA and RIA in synovial fluid from the knee joints of patients with RA and in healthy control subjects. Values are medians (and Q_1_–Q_3_). *P *relates to long-standing RA versus early RA (Mann–Whitney test). ^a^*n *= 7; ^b^*n *= 6; ^c^*n *= 28; ^d^*n *= 23; ^e^*n *= 25; ^f^*n *= 2; ^g^n = 10. BN/GRP, bombesin/gastrin-releasing peptide; CGRP, calcitonin-gene-related peptide; MCP-1, monocyte chemoattractant protein-1; nd, not detectable; nm, not measured; NPY, neuropeptide Y; ns, not significant; RA, rheumatoid arthritis; SP, substance P; TNF, tumour necrosis factor; VIP, vasoactive intestinal peptide.

**Table 2:** Concentrations of sTNFR1 and neuropeptides in serum

Variable	Early RA (pg/ml)	Long-standing RA (pg/ml)	*P*	Controls (pg/ml)
sTNFR1	1500^a^	1900^b^	ns	1200^d^
	(1300–1700)	(1600–2000)		
BN/GRP	3000^a^	1700^b^	<0.05	340^e^
	(2400–3300)	(430–2500)		
CGRP	1500^a^	820^b^	ns	310^e^
	(1100–1600)	(510–1600)		
NPY	610^a^	900^c^	ns	930^f^
	(370–890)	(700–1900)		
SP	170^a^	410^b^	<0.05	180^g^
	(150–300)	(320–440)		
VIP	250^a^	280^b^	ns	290^g^
	(210–280)	(230–340)		
ESR	20^a^	32^b^	ns	nm
	(7 – 32)	(22 – 47)		

Concentrations were measured using ELISA. Values are medians (and Q_1_–Q_3_). The ESR is also shown. *P *relates to long-standing RA versus early RA. ^a^*n *= 4; ^b^*n *= 22; ^c^*n *= 21; ^d^sTNFR1 value in healthy controls according to the manufacturer; ^e^*n *= 4; ^f^*n *= 2; ^g^*n *= 11. BN/GRP, bombesin/gastrin-releasing peptide; CGRP, calcitonin-gene-related peptide; ESR, erythrocyte sedimentation rate; NPY, neuropeptide Y; nm, not measured; ns, not significant; RA, rheumatoid arthritis; SP, substance P; sTNFR1, soluble tumour necrosis factor receptor 1; VIP, vasoactive intestinal peptide.

**Table 3:** Factor analysis on cytokines and neuropeptides in synovial fluid

Variable	Factor			
	
	1	2	3	4
TNF-α		0.751		
IL-6			0.913	
MCP-1		0.912		
BN/GRP	-0.436		0.623	0.327
SP	0.409	0.33	0.361	0.671
VIP	0.784			
CGRP	0.715			-0.472
NPY	0.893			
ESR				0.71

Principal-component analysis for cytokines and neuropeptides in synovial fluid from 23 patients with long-standing RA, and for the ESR. BN/GRP, bombesin/gastrin-releasing peptide; CGRP, calcitonin-gene-related peptide; ESR, erythrocyte sedimentation rate; MCP, monocyte chemoattractant protein-1; NPY, neuropeptide Y; RA, rheumatoid arthritis; SP, substance P; TNF, tumour necrosis factor; VIP, vasoactive intestinal peptide.

**Table 4:** Factor analysis on sTNFR1 and neuropeptides in serum

Variable	Factor		
	
	1	2	3
sTNFR1		0.323	0.827
BN/GRP	0.324	0.778	
SP	0.907		
VIP	0.934		
CGRP	0.63	0.575	
NPY	0.925		
ESR			0.897

Principal-component analysis for sTNFR1 and five neuropeptides in serum from 21 patients with long-standing RA, and for the ESR. BN/GRP, bombesin/gastrin-releasing peptide; CGRP, calcitonin-gene-related peptide; ESR, erythrocyte sedimentation rate; NPY, neuropeptide Y; RA, rheumatoid arthritis; SP, substance P; sTNFR1, soluble tumour necrosis factor receptor 1; VIP, vasoactive intestinal peptide.
